# Microbes on the “peachy spots” of ancient Kaihua paper: microbial community and functional analysis

**DOI:** 10.3389/fmicb.2023.1326835

**Published:** 2024-01-11

**Authors:** Yanjun Zhou, Yan Shi, Yanyan Huang, Jiang Zhong

**Affiliations:** ^1^Department of Microbiology and Microbial Engineering, School of Life Sciences, Fudan University, Shanghai, China; ^2^Institute for Preservation and Conservation of Chinese Ancient Books, Fudan University, Shanghai, China

**Keywords:** microbial community, traditional Chinese handmade paper, biodeterioration, Kaihua paper, functional prediction

## Abstract

Kaihua paper is a type of precious hand-made paper in China that has been used throughout Chinese history. Due to its extraordinary whiteness and fine texture, it was adopted by the imperial palace in the reign of Emperor Kang Xi and Yong Zheng of the Qing Dynasty of China in the 17th and 18th century. It is stained by a special type of yellowish-brown spot after years of storage, which is called a “peachy spot.” The formation of such spots remains unclear, although complicated physicochemical processes or microbial activities might be involved. We performed nondestructive sampling and high-throughput sequencing on peachy spot surfaces, unstained areas, and air samples in the stack room to analysis the the bacterial and fungal communities, and performed prediction of functional genes of the bacterial communities. The results showed that peachy spot formation was mainly related to bacterial communities rather than fungal communities. Significantly more potential acid- producing, acidophilic or cellulase-producing bacteria, such as, *Streptococcus*, *Staphylococcus*, and *Lysinibacillus*, and pigment-producing bacteria, such as *Methylobacterium* and *Rubrobacter*, were identified in the peachy spot samples. Prediction of the functional genes of the bacterial community also suggested the production of acidic substance pigments. These findings provide new insights into the pigment formation mechanism in ancient paper and open an opportunity to develop new strategies to preserve the ancient paper documents.

## 1 Introduction

Paper, as a carrier of human thought, art and history, is critical in human society. Paper relics, like all other cultural heritage materials, are nonrenewable and will degenerate over time. Thus, preserving paper relics is a cultural and technological challenge.

Microbial deterioration of paper materials has always been a focus in the preservation of paper relics (Cappitelli et al., 2010). Early studies on the biodegradation of cultural heritage have been carried out for approximately a century (Sterflinger et al., 2018), using mostly culture-dependent methods and microscopic observations (Mitchell and McNamara, 2010). Fungi, especially filamentous fungi, are considered the most important organisms in microbial hazards. Due to technical limitations, only culturable microbes, which represent only a small part of the total microbes, were studied (Amann et al., 1995). These culturable strains were probably not the dominant microbes that caused paper damage. In recent years, new methods have been applied to study the microbial community on various historical relics, including paper, parchment, storeroom objects, and photographic materials (Sclocchi et al., 2016; Migliore et al., 2017; Liu et al., 2018; Piñar et al., 2020; Branysova et al., 2022). Genomic analysis based on high-throughput sequencing is becoming a powerful approach to study the diversity and impact of microorganisms on historical objects (Kraková et al., 2018; Sterflinger et al., 2018; Beata, 2020).

Traditional Chinese handmade paper was made of plant fiber, following slightly different processes depending on the geographical area and historical period of production. The sources of plant fiber include bark, straw, and bamboo. Moreover, paper is often coated with gelatin or other substances to make it impermeable to ink (Area and Cheradame, 2011). These diverse organic and inorganic components in paper provide substrates for microbial growth and reproduction under favorable conditions, which in turn destroys the structure of the paper (Sterflinger, 2010). Therefore, different types of paper may favor different microorganisms that settle on its surface, resulting in different structures of microbial communities and ultimately different kinds of damage.

Kaihua paper is a unique type of ancient paper preserved to this day that is famous for its delicate and white and thin characteristics. It was one of the most valuable royal papers in the Qing Dynasty, especially during the reign of the three emperors, Kangxi, Yongzheng and Qianlong (1662–1795 A.D.). The plant fiber used to make Kaihua paper was from the family *Thymelaeaceae*. Ancient Kaihua paper commonly developed isolated or coalescent yellowish-brown spots, which are called “peachy spots.” Such spots affect the paper greatly and may lead to irreversible loss of historical content. Morphologically, peachy spots resemble the well-studied “foxing spots,” a common damage to paper cultural relics (Meynell and Newsam, 1978). Among the many hypotheses about foxing formation, metals and microorganisms are considered the two major causes (Choi, 2007). However, the role of microorganisms in the formation of “peachy spots” on Kaihua paper remains unknown.

In this study, culture-independent high-throughput sequencing was used to identify bacterial and fungal communities on Kaihua paper affected by peachy spots. The potential roles of bacterial and fungal groups in the formation of peachy spots were explored by correlation analysis and prediction of functional genes of the microbial community.

## 2 Materials and methods

### 2.1 Tested paper samples

**Figure 1:**
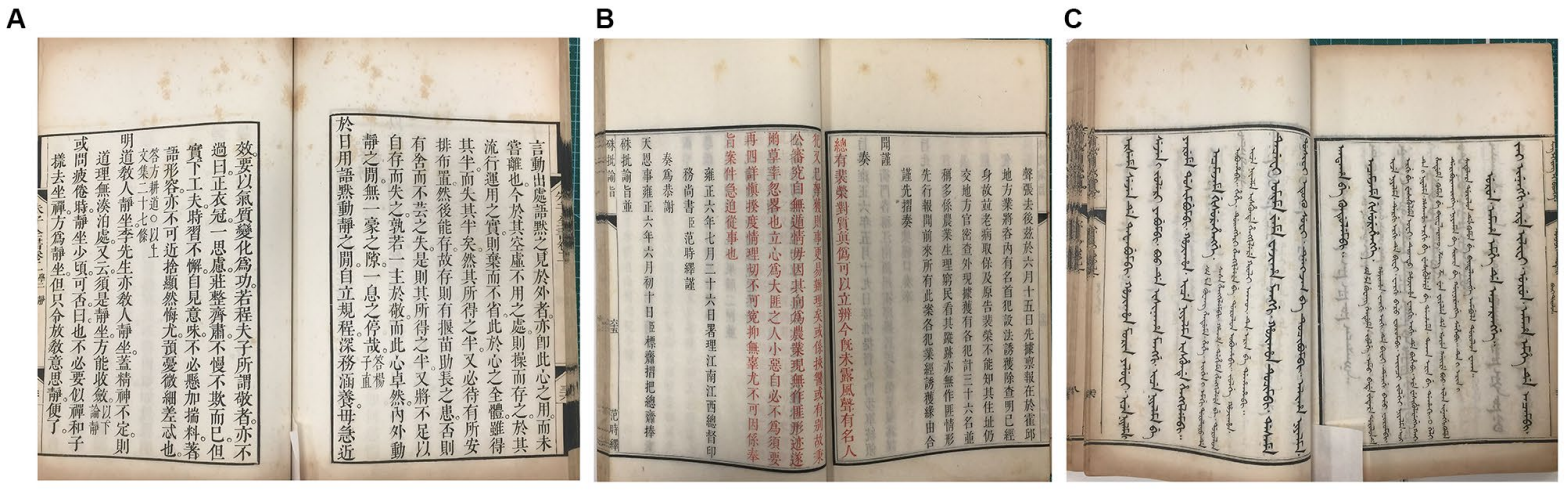
Representative images of the “peachy spots” of Kaihua paper from three sets of ancient books. **(A)** *Zhu Zi Quan Shu* dated approximately 1713–1717 A.D. **(B)** *Zhu Pi Yu Zhi* dated approximately 1713–1717 A.D. **(C)** *Yu Zhuan Xing Li Jing Yi* in Manchu dated approximately 1732–1738 A.D.

Three sets of ancient books made of Kaihua paper preserved in Fudan University Library were selected for this study (Figure 1). These books came from the reign of Emperor Kang Xi and Yong Zheng and Qian Long of the Qing Dynasty of China in the 18th century and have been kept in the same stack room for rare ancient books for a long time without being touched. The temperature of the stack room is kept at 16 ~ 20°C and the humidity at 50 ~ 60%. Although the books are in relatively good condition, there are obvious peachy spots on each set of books.

### 2.2 Sampling method

The sampling areas most suitable for the study were chosen and evaluated carefully, and the sampling protocol was designed to minimize impact to the paper. A sterilized polyester fiber swab (SwwipTM, Cleanmo, Shenzhen, China) was selected as the sampling tool (swab head size: 4.0 mm x 11.0 mm, total length: 70.0 mm). The swab head was fully moistened with 20 μL of sterilized wetting solution (0.15 M NaCl and 0.1% Tween-80, dissolved in ultrapure water) and used to gently wipe the preselected sampling position 20 times to collect the microorganisms on the paper surface. Then, the swab head was peeled off using sterile tweezers and immediately transferred into the PowerBead extraction tube in the MOBIO PowerSoil DNA Isolation Kit (Mo Bio, Carlsbad, CA, USA). For each set of books, three samples of peachy spots and one sample of unstained area of the inner page were collected.

To exclude interference from airborne microorganisms of the stack room at the time of sampling, the indoor air sedimentation sample were independently collected as the control. Three sterilized polyester fiber swabs were moistened as other sample swabs and allowed to stand on the sampling bench during the sampling, which lasted about 1 h. At the end of the sampling, the heads of three swabs were peeled off and transferred into a single extraction tube for DNA extraction and amplification process, following the same procedure as the other samples to keep consistency.

Samples were stored in a − 20°C freezer, and DNA extraction was performed within 12 h.

### 2.3 DNA extraction and sequencing

For DNA extraction, each sample was processed using the MOBIO PowerSoil DNA Isolation Kit following the manufacturer’s instructions with minor modifications. The C6 solution provided by the kit was replaced with sterile TE buffer to minimize DNA degradation.

Sequences of the 16S rDNA and ITS rDNA were amplified for bacteria and fungi, respectively, using primer sets of 515F (GTGCCAGCMGCCGCGGTAA)/806R (GGACTACHVGGGTWTCTAAT) for the V4 region of 16S rDNA (Kuczynski et al., 2011) and ITS5-1737F (GGAAGTAAAAGTCGTAACAAGG)/ITS2-2043R (GCTGCGTTCTTCATCGATGC) for region 1 of ITS rDNA (Bellemain et al., 2010). The amplification was conducted according to the following program: initial denaturation at 94°C for 5 min, 20 cycles of denaturation at 94°C for 45 s, annealing at 50°C for 30 s, and extension for 90 s at 72°C, final extension at 72°C for 10 min. Then the same amplification was performed once again to add the barcode sequences to the primers for sample distinction after sequencing.

The concentration, total amount and purity of the amplification products were determined, and then the qualified amplification products were sequenced on the Illumina MiSeq (300-bp paired-end reads) platform (Illumina Inc., San Diego, United States) by Novogene Bioinformatics Technology Co., Ltd. (Beijing, China).

### 2.4 Data processing

The QIIME platform (Caporaso et al., 2010) was used to filter the acquired sequences to remove low quality (Q value less than 20), short length (shorter than 250 bp) and chimeric sequences. The sequences were split according to their taxonomy and assigned to operational taxonomic units (OTUs) at the 97% similarity level using MOTHUR^1^ (Schloss et al., 2009). The representative sequences of these OTUs were classified within the SILVA database release 119 for bacteria and UNITE for fungi (Abarenkov et al., 2010).

### 2.5 Microbial community analyses and functional gene prediction

The QIIME platform was used to analyze the bacterial and fungal community structure and composition on the normalized OTU dataset. Rarefaction curves were created to assess sampling efficiency. Three alpha diversity indexes, Chao 1, PD value and Shannon index, were calculated to reflect the overall diversity and complexity of the microbial community.

Multivariate analyses on the OTU dataset were performed using the UniFrac unweighted metric in QIIME. Principal coordinates analysis (PCoA) was performed to show the differences between the sample sets. Species abundance clustering heatmaps were used to analyze the microbial composition structure.

Tax4Fun was implemented to predict the functional differences between unstained areas and peachy spots. Tax4Fun is an R package that links 16S rRNA gene sequences with the functional annotation of sequenced prokaryotic genomes, which is realized with a nearest-neighbor identification based on a minimum 16S rRNA sequence similarity (Aßhauer et al., 2015). It was used to predict the functional genes from the 16S rDNA information of “peachy spots” groups and samples from unstained area based on the Silva database, and the KEGG functional annotation abundance table was obtained. Then STAMP software (Parks et al., 2014) was used to screen for genes with significant differences in abundance between “peachy spots” samples and unstained samples. Only differences with *p* values less than 0.05 were reported.

### 2.6 Statistical analyses

All statistical analyses were conducted in the R environment (v3.4.3; http://www.r-project.org/), and all statistical tests were considered significant at p values less than 0.05. Two-way nested ANOSIM (analysis of similarity) was used to test for significant differences in microbial composition (Clarke and Warwick, 1994). Wilcoxon’s test was used to compare the relative abundance between the unstained areas and peachy spots.

## 3 Results

### 3.1 Sequencing quality, rarefaction curve and alpha diversity

Samples were taken from the surface of three sets of ancient books made of Kaihua paper: Zhu Zi Quan Shu, Zhu Pi Yu Zhi, and Yu Zhuan Xing Li Jing Yi in Manchu. Sequencing of the amplicon libraries of 16S rDNA and ITS rDNA yielded a total of 1,656,407 raw reads belonging to bacteria and fungi. After quality trimming and assigning reads to their respective samples, 789,811 high-quality 16S rDNA sequences and 810,781 high-quality ITS rDNA sequences were obtained for 13 samples, including 9 samples of peachy spots, 3 samples of unstained areas, and 1 stack room air sample (Table 1).

**Table 1:** Summary of sample information and high-quality sequences.

Group	Sample	Origin	Sampling area	High-quality sequence (16S)	High-quality sequence (ITS)
KTa	KTa1	Zhu Zi Quan Shu	“peachy spots”	84,153	73,858
	KTa2	Zhu Zi Quan Shu	“peachy spots”	62,210	75,413
	KTa3	Zhu Zi Quan Shu	“peachy spots”	51,495	62,134
KTb	KTb1	Zhu Pi Yu Zhi	“peachy spots”	69,066	51,474
	KTb2	Zhu Pi Yu Zhi	“peachy spots”	53,649	55,645
	KTb3	Zhu Pi Yu Zhi	“peachy spots”	54,788	54,764
KTc	KTc1	Yu Zhuan Xing Li Jing Yi in Manchu	“peachy spots”	68,239	48,333
	KTc2	Yu Zhuan Xing Li Jing Yi in Manchu	“peachy spots”	74,378	74,693
	KTc3	Yu Zhuan Xing Li Jing Yi in Manchu	“peachy spots”	25,497	69,569
K	Ka	Zhu Zi Quan Shu	unstained area	67,128	59,748
	Kb	Zhu Pi Yu Zhi	unstained area	84,958	83,747
	Kc	Yu Zhuan Xing Li Jing Yi in Manchu	unstained area	50,844	50,514
Control	Control		indoor air	43,406	50,889

**Figure 2:**
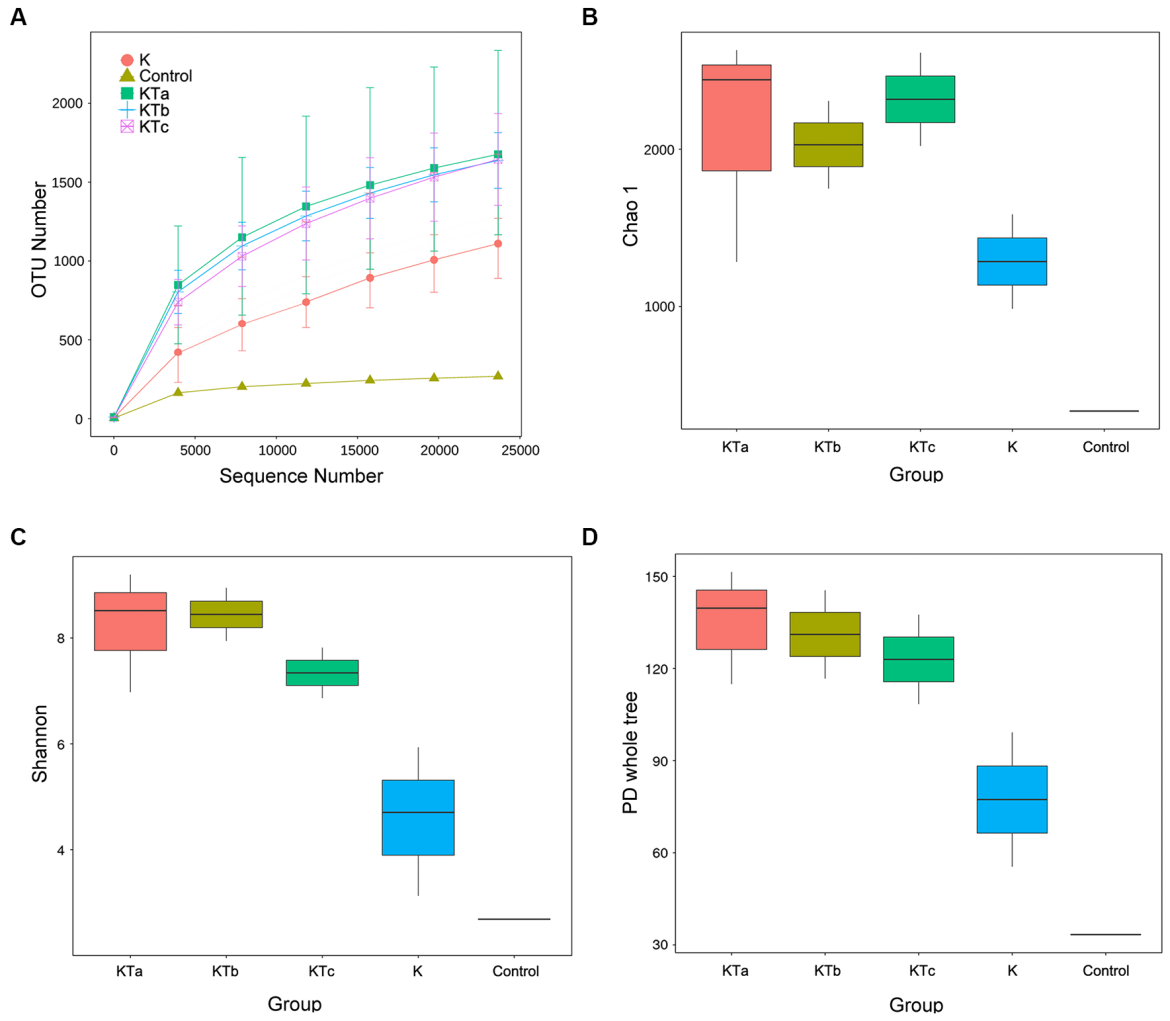
Rarefaction curves and alpha diversity index of bacterial communities of 9 “peachy spot” samples, 3 unstained samples and 1 air sample. **(A)** Rarefaction curves; **(B)** Chao1 index; **(C)** Shannon index; **(D)** PD value. The alpha diversity of the KT group (KTa, KTb and KTc) was significantly higher than that of the K group (*p* < 0.05).

**Figure 3:**
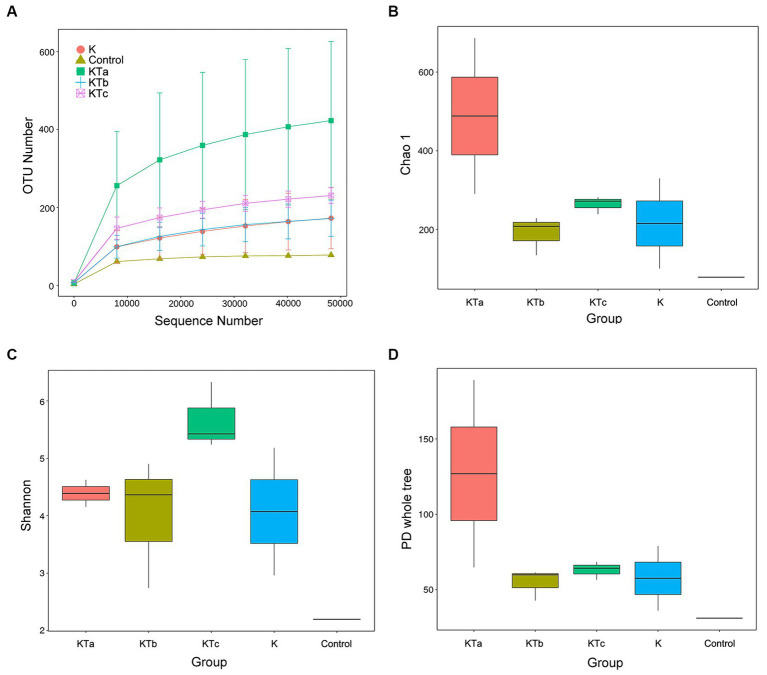
Rarefaction curves and alpha diversity index of fungal communities of 9 “peachy spot” samples, 3 unstained samples and 1 air sample. **(A)** Rarefaction curves; **(B)** Chao1 index; **(C)** Shannon index; **(D)** PD value.

The rarefaction curves confirmed the efficiency of the sampling (Figures 2A, 3A). In the bacterial part, the alpha diversity index of the peachy spots was significantly higher than that of the unstained group (*p* < 0.05), and the alpha diversity indexes of the three groups of peachy spot samples were very close to each other, with no significant differences. The diversity of the bacterial community at the peachy spots was significantly higher than that of the unstained area of the same book (Figures 2B–D). However, no consistent difference was found in the diversity of the fungal community between peachy spots and unstained areas (Figures 3B–D). In addition, there was a significant within-group difference in the Chao1 index and PD value in the KTa group (Figures 3B,D), indicating that the fungal community structure varied greatly between spots in the same book. It seemed that peachy spots on Kaihua paper were more related to bacteria than fungi.

### 3.2 Microbial community structure on the “peachy spots”

Species annotation was performed on all bacterial and fungal OTUs. Most identified bacteria belonged to the phyla *Bacillota*, *Pseudomonadota*, and *Actinomycetota* (Supplementary Table S1). All identified fungi belonged to 7 phyla. The phylum *Ascomycota* occurred with the highest abundance in all samples, followed by *Basidiomycota* and *Zygomycota* (Supplementary Table S2).

**Figure 4:**
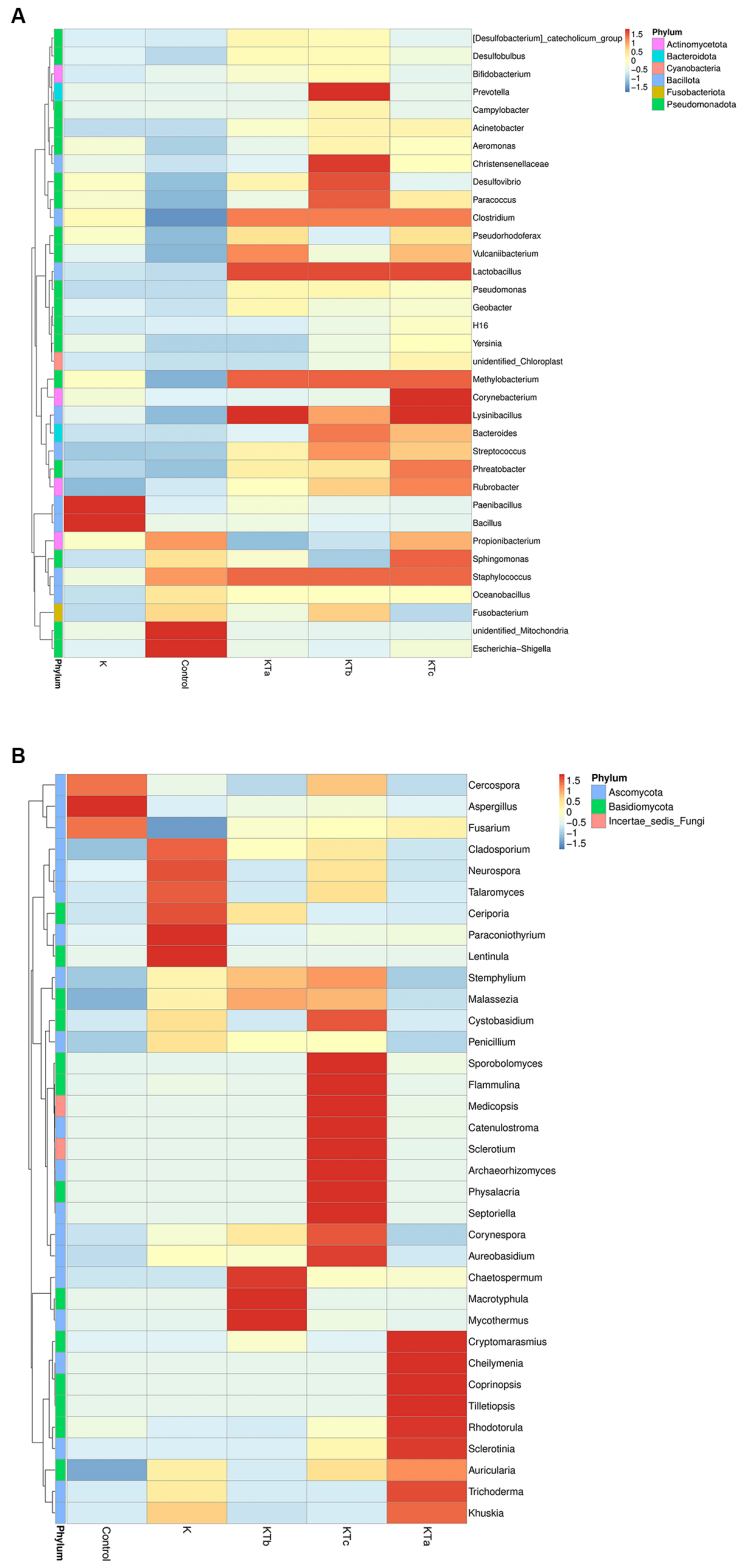
Heatmap of grouping clustering in bacterial communities **(A)** and fungal communities **(B)**. Thirty-five genera with the highest proportion were used for this cluster analysis.

The top 35 most abundant genera of either bacteria or fungi were used for cluster analysis (Figure 4). The heatmap showed that the bacterial communities of high abundance were quite similar within peachy spot groups KTa, KTb, and KTc and were clearly different from the unstained group (K group) and the air deposition sample (control) (Figure 4A). Peachy spot groups had significantly higher proportions of *Bacillota*, such as *Methylobacterium*, *Christensenellaceae*, *Streptococcus*, *Lysinibacillus*, *Staphylococcus* and *Lactobacillus*, than the unstained group and air deposition sample (Figure 4A). In addition, *Rubrobacter* of *Actinobacteria* was also significantly aggregated in peachy spots, whereas the proportion of other genera of *Actinobacteria* and *Pseudomonadota* was relatively low (Figure 4A). Among the unstained paper samples, *Paenibacillus* and *Bacillus* were more prominent (Figure 4A). In the air deposition sample, there were significantly more *Escherichia-Shigella* (Figure 4A).

Furthermore, the clustering of the species abundance of fungi varied greatly. No significant similarity was found between different peachy spot groups or between peachy spot groups, the unstained paper group, and the air deposition sample (Figure 4B), indicating that fungi might not be the main cause of peachy spots. *Aspergillus*, which was frequently found on ancient books, aggregated in the air sample but not in the paper samples (Figure 4B). In addition, the proportions of *Cercospora* and *Fusarium* were also higher in the air deposition sample than in all paper samples (Figure 4B). In the unstained paper group, there were high abundances of *Cladosporium*, *Neurospora* and *Talaromyces* (Figure 4B). These genera include multiple species that are common plant endophytes.

**Figure 5:**
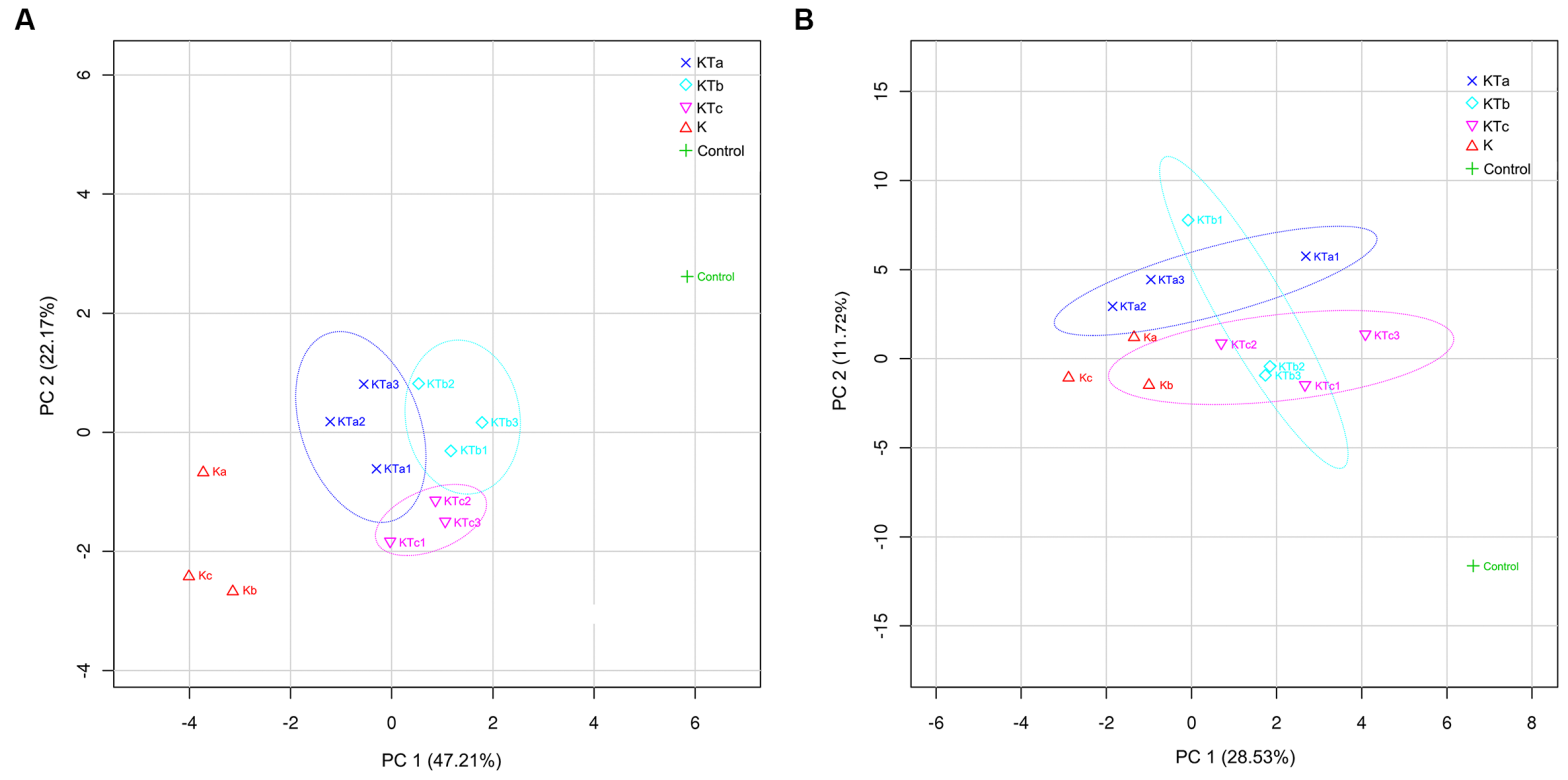
PLS-DA (partial least squares discrimination analysis) of bacterial communities **(A)** and fungal communities **(B)**.

To reveal the differences in community structure between peachy spot areas and unstained areas, we conducted PLS-DA to compare the composition of both bacteria and fungi in all samples. The results showed that the microbial community (both bacteria and fungi) on the paper surface could be completely separated from that of the indoor air of the stack room (Figure 5). The bacterial community of peachy spot samples could also be separated from the unstained paper samples (Figure 5A), whereas the fungal community of these two sets of samples overlapped (Figure 5B). Moreover, the bacterial community structures of the peachy spot groups were relatively close to each other, and the difference within each group was less than that of fungi (Figure 5). These results also suggested that the formation of peachy spots may be directly related to the bacterial community but not to the fungal community.

### 3.3 Functional gene prediction

To elucidate the potential contribution of the bacteria to the formation of peachy spots, we defined the peachy spot samples in all books as the peachy spot group (KT) and the samples from unstained area of the inner page in all books as the unstained paper group (K). The R package Tax4Fun was used to predict functional genes in the bacterial community from the 16S rDNA data of the two groups. STAMP was used to screen for genes with significant differences in abundance between groups.

**Figure 6:**
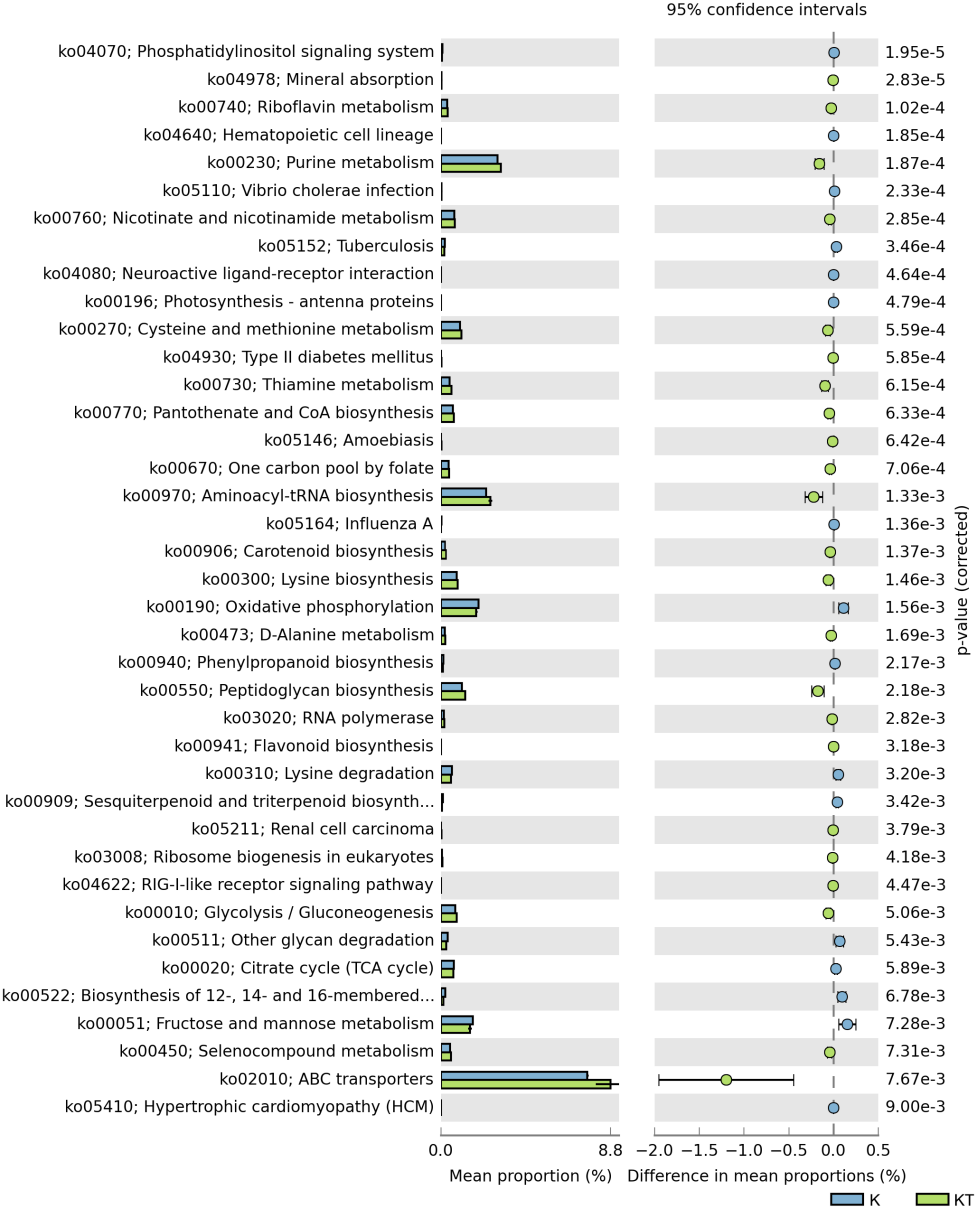
Predicted KEGG ortholog groups with significant differences (*p* < 0.05) in relative abundance between the “peachy spots” and unstained paper.

The abundance of some gene ortholog groups, such as mineral absorption, riboflavin metabolism, carotenoid biosynthesis, and lysine biosynthesis, was significantly higher in the peachy spot group than in the unstained paper group (Figure 6). This also suggested that the bacterial community at the peachy spots area might be associated with the accumulation of pigments and the increased production of acidic substances.

## 4 Discussion

Ancient paper was made of many organic and inorganic compounds that can be used by bacteria and fungi. Traditionally, groups of fungi, including *Penicillium*, *Aspergillus*, *Trichoderma*, *Chaetomium* and *Alternaria*, were considered important for paper damage (Arai, 2000; Nitiu et al., 2020), as they are relatively easy to observe and culture. However, they may not necessarily be the key microbes that cause paper damage.

Most previous studies have also linked fungi to the cause of paper stains. Microscopic observation of the presence of mycelia has been used as a criterion to determine whether a stain is due to mold or mildew damage. The presence of UV fluorescence is used as a standard to define “foxing spots” (Meynell and Newsam, 1978; Koochakzaei and Alizadeh Gharetapeh, 2021), but fluorescence is also considered by other researchers as a factor in the formation of “foxing” spots (Bicchieri et al., 2001; Figueira et al., 2020). The peachy spots on the Kaihua paper resemble foxing spots by description, although in our previous tests, no UV fluorescence was observation at the spots (data not shown).

In this study, we used high-throughput sequencing technology to determine the bacterial and fungal communities on the surface of peachy spots and compared them with unstained surfaces as well as air samples from a stack room. The results showed that the abundance of *Penicillium* was not significantly higher in the position of peachy spots than that without it. The abundance of *Aspergillus* in the air deposition sample of the stack room was even higher than that of peachy spot samples. This implied that these typical filamentous fungi might have come from the stack room environment where the ancient books were stored but had not stably established on the surface of Kaihua paper and thus were not related to the formation of peachy spots. *Cercospora* and *Fusarium* were also found to be relatively abundant in the air deposition sample, both of which have multiple species that are pathogenic to plants (Daub and Ehrenshaft, 2000; Rampersad, 2020; Sinha et al., 2023). These fungi may come from other contaminated ancient books or old wooden bookshelves and furniture and pose a potential threat to paper (Şimşekli et al., 1999; Gravesen, 2000; Piñar et al., 2013). In addition, high abundances of *Cladosporium*, *Neurospora* and *Talaromyces* were found in the unstained paper samples, and multiple species of these genera belong to plant endophytes (Tian et al., 2009; Yamagiwa et al., 2011; Nitiu et al., 2020).

The results suggested that the formation of peachy spots on Kaihua paper might be more related to bacteria than fungi. The role of bacteria in the conservation and preservation of paper relics has long been neglected or underestimated. In recent years, more attention has been given to bacterial communities, where the bacterial genera *Bacillus*, *Clostridium*, *Pseudomonas*, *Micrococcus*, *Actinomyces* and *Cellulophaga* were often found to be related to paper damage (Kraková et al., 2012; Dunca et al., 2014; Lech, 2016; Szulc et al., 2018; Raeisnia et al., 2022; Pavlović et al., 2023). In this study, we found significantly more *Actinomycetes* of the genus *Rubrobacter* in peachy spot groups. Species of the genus *Rubrobacter* has previously isolated from deteriorated monuments (Piñar et al., 2013) and the carotenoid pigments they produce has been found to have a causal relationship with the rosy discoloration of ancient wall paintings (Imperi et al., 2007; Ettenauer et al., 2014). And *Actinomycetes* have been reported to possibly utilize insoluble matrix components and penetrate materials in parchment and canvas paintings and even increase the water solubility of the matrix for further utilization (Pavić et al., 2015; Lech, 2016).

Nevertheless, other bacteria commonly present on paper relics were not abundant (below 1% for *Clostridium* and *Pseudomonas*) or even absent (*Micrococcus* and *Cellobacteria*) in peachy spot samples. Instead, bacteria with acid-producing, acidophilic or cellulase-producing potential, including *Lactobacillus*, *Streptococcus*, *Staphylococcus*, and *Lysinibacillus*, and bacteria with pigment-producing potential, such as *Methylobacterium* (van Aken et al., 2004; Vadivukkarasi et al., 2018), were found to be highly abundant in peachy spots.

The result of functional gene prediction with Tax4fun supported the hypothesis regarding the role of bacteria in peachy spot formation. The abundance of predicted bacterial gene orthologs involved in the biosynthesis and metabolism of riboflavin, carotenoids and flavonoids was significantly higher in the peachy spot groups than in the unstained paper group. Some bacteria have the capability to produce organic acids and various enzymes to hydrolyze the paper components (Pavić et al., 2015), making the material easier to be colonized by more microbes, and the addition of pigment-producing bacteria may further help the stain formation. It is also possible that the chemical composition of peachy spot areas promoted the survival of certain bacteria and shaped the structure of the bacterial community.

The results obtained in the present study also showed that bacteria and fungi not only coexist in the microenvironment of the paper surface but also form relatively stable communities in each group. This may be because the paper itself continues to exert selective pressure on microorganisms. Studies of purple spots on ancient parchment hypothesized a succession model, starting from halophilic archaea, followed by halotolerant bacteria and actinomycetes, and then fungi (Migliore et al., 2019). Similarly, peachy spots on Kaihua paper may be the result of synergy and competition between different kinds of archaea, bacteria, and fungi. Their succession caused stains and the aging of Kaihua paper after a long time. Some archaea have been found to produce pink pigments in previous study (Mazzoli et al., 2018; Tichy et al., 2023). Although archaea were not investigated in this study, they could have played a role in the biodeterioration of Kaihua paper. The accumulation of bacteria with potential acid- and pigment-producing ability may be a relatively late phenomenon in the process of peachy spot formation. The results also remind us that without timely intervention, the bacteria at peachy spots will continue to destroy precious cultural relics.

Although studies based on DNA sequencing provide clues for the peachy spots, they may not tell the current microbial activities on the paper. Part of the detected DNA may be the remains of dead cells that have not been degraded completely. This gives us an opportunity to investigate the microbial community involved in the formation of peachy spots, but it also increases the difficulty of analyzing the process. One solution is to analyze RNA, which is much less stable than DNA. However, we found that the concentration of RNA recovered from the paper surface using noninvasive methods was too low to study. Due to the limitation of sample numbers and the lack of integrated analysis of multiple omics, the exact mechanism of peachy spot formation needs to be further studied.

In summary, through nondestructive sampling and high-throughput sequencing, it was found that the bacterial community on the peachy spots on Kaihua paper significantly differed from that on the unstained paper and the air in the stack room. No such difference was found in the fungal community. Bioinformatics analysis and functional gene prediction indicated that the formation of peachy spots may be mainly related to bacteria rather than fungi. The bacterial community may be mostly responsible for acidic substance and pigment accumulation in paper. The mechanisms of peachy spot formation are rather complex, and therefore, an increase in sample size and multiomics joint analysis will be useful to reveal the key microorganisms and their succession process that cause this phenomenon.

## Data availability statement

The datasets presented in this study can be found in online repositories. The names of the repository/repositories and accession number(s) can be found below: NCBI, PRJNA1031546, PRJNA1032408.

## Author contributions

YZ: Data curation, Investigation, Visualization, Writing – original draft. YS: Data curation, Writing – review & editing. YH: Investigation, Methodology, Writing – original draft. JZ: Conceptualization, Funding acquisition, Resources, Supervision, Writing – review & editing.

## Footnotes

^1^ http://www.mothur.org/
