# Supplementary material for: Microbes on the “peachy spots” of ancient Kaihua paper: microbial community and functional analysis
Source: Front Microbiol. 2024 Jan 11;14:1326835. doi: 10.3389/fmicb.2023.1326835 (PMC10808800; doi:10.3389/fmicb.2023.1326835)
Supplement: Supplementary file 1 [file Data_Sheet_1.docx]

Supplementary Material

Microbes on the “peachy spots” of ancient Kaihua paper: microbial community and functional analysis

Yanjun Zhou^1^, Yan Shi^1^, Yanyan Huang^2^, Jiang Zhong^1,2*^

# 1 Supplementary Tables and Figures

## 1.1 Supplementary Tables

**Supplementary Table 1.** Distribution of the top 10 phyla of bacteria in relative abundance in "peachy spots", unstained paper and indoor air.

| Phylum | KTa | KTb | KTc | K | Control |
| --- | --- | --- | --- | --- | --- |
| *Bacillota* | 21.48% | 23.54% | 21.38% | 53.46% | 11.61% |
| *Pseudomonadota* | 47.33% | 44.05% | 52.30% | 30.65% | 81.77% |
| *Actinomycetota* | 10.30% | 8.66% | 8.47% | 5.41% | 2.80% |
| *Bacteroidota* | 3.86% | 8.44% | 5.30% | 2.54% | 0.92% |
| *Acidobacteriota* | 4.32% | 3.52% | 2.67% | 0.93% | 0.06% |
| *Nitrospirota* | 1.66% | 0.21% | 0.61% | 0.50% | 0.02% |
| *Chloroflexota* | 1.51% | 0.97% | 0.88% | 0.72% | 0.20% |
| *Planctomycetota* | 1.50% | 1.34% | 1.35% | 1.00% | 0.12% |
| *Cyanobacteria* | 1.09% | 0.73% | 0.49% | 0.10% | 0.04% |
| *Verrucomicrobiota* | 1.49% | 1.14% | 1.22% | 0.76% | 0.15% |
| Others | 5.46% | 7.40% | 5.33% | 3.94% | 2.31% |

**Supplementary** **Table 2.** Distribution of fungal phyla in relative abundance in "peachy spots", unstained paper and indoor air.

| Phylum | KTa | KTb | KTc | K | Control |
| --- | --- | --- | --- | --- | --- |
| *Ascomycota* | 75.32% | 51.35% | 70.63% | 53.05% | 96.68% |
| *Basidiomycota* | 22.39% | 48.32% | 26.70% | 46.39% | 2.72% |
| *Zygomycota* | 2.11% | 0.25% | 1.27% | 0.00% | 0.39% |
| Incertae_sedis_Fung*i* | 0.01% | 0.01% | 1.21% | 0.01% | 0.00% |
| *Chytridiomycota* | 0.04% | 0.00% | 0.02% | 0.54% | 0.11% |
| *Neocallimastigomycota* | 0.02% | 0.08% | 0.13% | 0.00% | 0.00% |
| *Glomeromycota* | 0.04% | 0.00% | 0.04% | 0.00% | 0.09% |
| Others | 0.07% | 0.00% | 0.00% | 0.01% | 0.00% |
